# Aspirin-Exacerbated Respiratory Disease Complicated by Eosinophilic Esophagitis: A Case Report

**DOI:** 10.7759/cureus.74384

**Published:** 2024-11-25

**Authors:** Eiichi Kakehi, Kazuhiko Kotani

**Affiliations:** 1 Department of General Medicine, Tottori Municipal Hospital, Tottori, JPN; 2 Division of Community and Family Medicine, Jichi Medical University, Shimotsuke, JPN

**Keywords:** aspirin-exacerbated respiratory disease, eosinophilic esophagitis, epigastralgia, non-steroidal anti-inflammatory drug, prostaglandin d2 (pgd2)

## Abstract

A 59-year-old woman developed sudden dyspnea after taking non-steroidal anti-inflammatory drugs (NSAIDs) for epigastralgia. She had a history of bronchial asthma after childbirth. Computed tomography showed bilateral peripheral bronchial wall thickening, lumen narrowing, obstruction, and circumferential lower esophageal mucosal edema. The patient was diagnosed with aspirin-exacerbated respiratory disease (AERD), a hypersensitivity reaction. Respiratory symptoms improved with intravenous dexamethasone. Endoscopy confirmed lower esophageal mucosal edema; mucosal biopsy detected eosinophilic infiltration, suggesting eosinophilic esophagitis (EoE). Although EoE is often diagnosed after AERD, the patient was simultaneously diagnosed with AERD and EoE after taking NSAIDs. Thus, EoE should be considered as a potential comorbidity when AERD develops after NSAID administration for abdominal symptoms.

## Introduction

Early analgesic use is recommended for acute abdominal pain, regardless of underlying causes. However, non-steroidal anti-inflammatory drugs (NSAIDs) are specifically indicated for certain diseases, such as ureteral stones and biliary colic [1]. Aspirin-exacerbated respiratory disease (AERD) is a key concern as a hypersensitivity reaction because that reaction is triggered by nearly all NSAIDs rather than aspirin alone [2]. Considering that AERD can cause fatal asthma attacks, clinicians must properly diagnose and treat the disease. It is essential to exercise caution and avoid misusing NSAIDs without considering a patient’s disease condition and medical history [2].

Eosinophilic esophagitis (EoE) is an antigen-mediated allergic disease of the esophagus [3]. Typical EoE symptoms include dysphagia and food impaction; a study of EoE in Japanese patients showed that these symptoms occurred in 52.9% of cases, whereas epigastralgia and abdominal pain occurred in 4.7% of cases [4]. In such scenarios, clinicians may administer NSAIDs to treat abdominal pain in patients with undiagnosed EoE [1].

EoE reportedly develops during AERD [5] or after aspirin desensitization therapy for AERD [6]. EoE has also been identified in patients with AERD before aspirin desensitization therapy [7]. Although these findings suggest a tight interplay between AERD and EoE, no specific clinical precautions have been indicated. This report describes a case in which AERD and EoE were simultaneously diagnosed.

## Case presentation

A 59-year-old woman presented to our hospital’s emergency department with dyspnea shortly after taking oral loxoprofen for epigastralgia. Two weeks prior, she had begun experiencing epigastralgia. She ingested over-the-counter stomach medicine containing methyl methionine sulfonium chloride as its main ingredient, but her abdominal symptoms did not improve. She also developed transient morning wheezing but did not seek medical attention because the wheezing spontaneously resolved. One week before attending our hospital, the patient visited her primary care physician for treatment of persistent epigastralgia. Her primary care physician suspected a gastric ulcer and thus prescribed a proton pump inhibitor, but the epigastralgia did not improve. Because the patient continued to display asthma symptoms, her primary care physician prescribed loxoprofen and inhaled corticosteroids. At around 9:00 PM on the same day, she took oral loxoprofen due to worsening epicardial pain. Soon thereafter, she developed respiratory distress and was transported to our hospital’s emergency department.

The patient’s medical history included a grass pollen allergy and allergic rhinitis treated with desloratadine. At approximately 30 years of age, she developed bronchial asthma after childbirth and required brief treatment. She had a history of nasal polyps at a previous otorhinolaryngology visit but was currently asymptomatic. She did not routinely take analgesics.

During clinical examination, the patient was alert and oriented. Her vital signs were as follows: blood pressure, 150/73 mmHg; pulse rate, 60 beats/min; body temperature, 36.5°C; respiratory rate, 22 breaths/min; and oxygen saturation, 96% on supplemental oxygen with 80% before oxygen administration. Chest auscultation revealed wheezing with normal heart sounds. Abdominal examination revealed epigastric tenderness but normal bowel sounds. Skin examination findings, such as angioedema or urticaria, were unremarkable.

In the emergency department, the patient received 1000 mg of intravenous acetaminophen as symptomatic therapy, which alleviated the abdominal pain but exacerbated respiratory distress and reduced oxygen saturation to 85%, even with an oxygen mask at 2 L/min; notably, it did not impact blood pressure. There were no accompanying skin symptoms or cardiovascular deterioration.

Laboratory tests revealed a peripheral blood eosinophil count of 560/µL (white blood cell count, 3600/µL) and biochemistry parameters within reference ranges. Coagulation studies showed no elevation of D-dimer. Electrocardiography demonstrated sinus rhythm without pathological changes.

**Figure 1:**
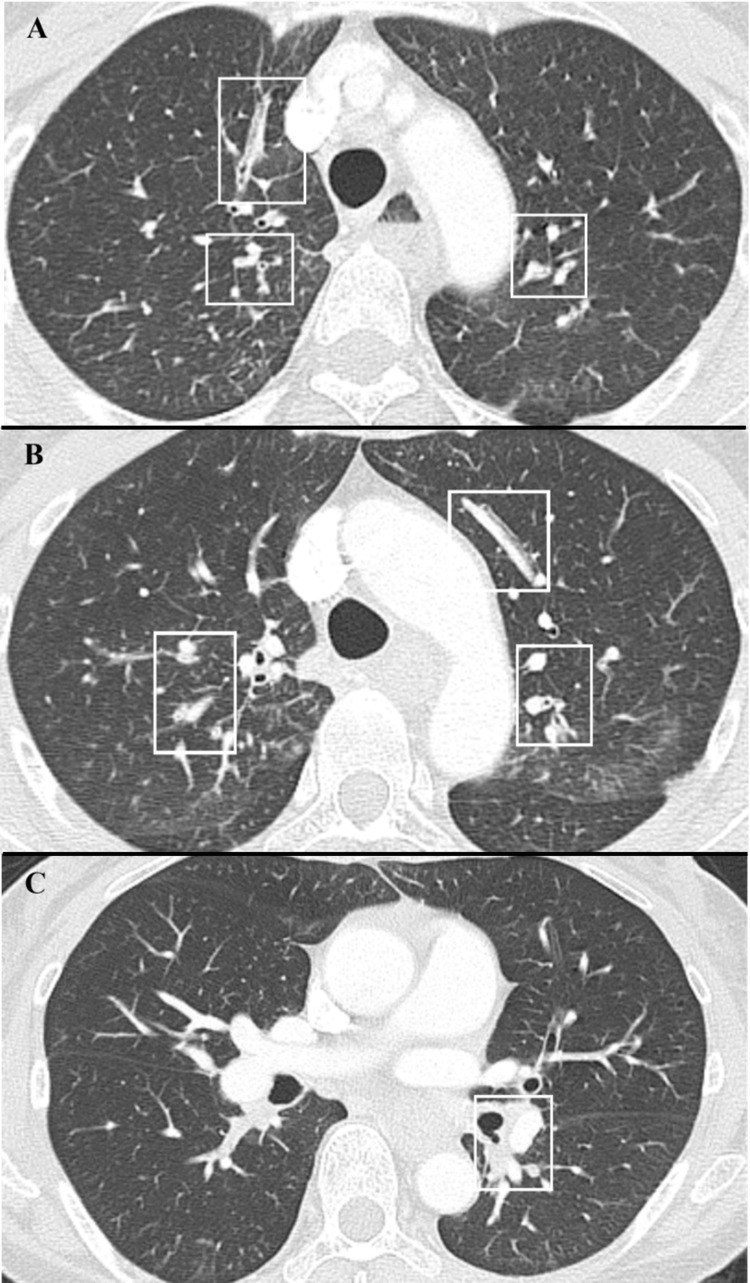
Computed tomography image of the chest Chest CT showed peripheral bronchial wall thickening, lumen narrowing, and obstruction in the bilateral upper lobes and left lower lobe (A, B, and C) (□).

**Figure 2:**
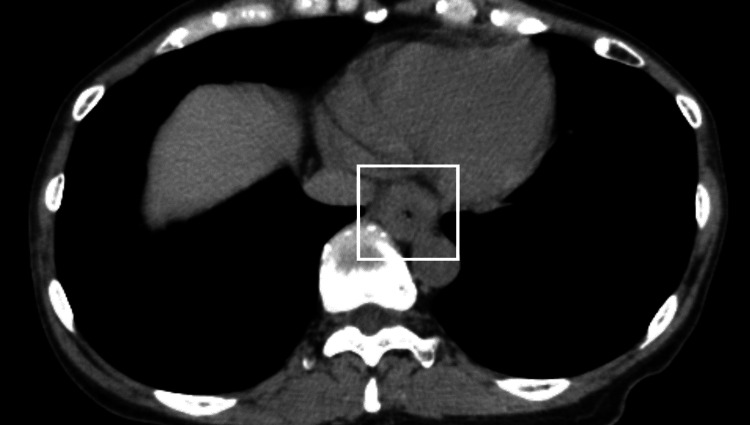
Chest computed tomography image of the lower esophagus Chest CT showed circumferential wall edema from the middle to the lower esophagus (□).

Subsequent chest computed tomography (CT) showed bilateral peripheral bronchial wall thickening, lumen narrowing, and obstruction in both upper lobes and the left lower lobe (Figure 1), along with circumferential wall edema from the middle to lower esophagus (Figure 2). Although anaphylaxis was initially considered in the differential diagnosis of acute wheezing, the criteria for anaphylaxis were not met [8]. Thus, the symptoms were attributed to bronchial asthma. The patient had a history of allergic diseases and bronchial asthma; past asthma episodes had been triggered by NSAID use. Based on these historical factors, she was diagnosed with AERD [2], but an aspirin challenge test was not conducted because of its potential impact on the patient’s health. She was treated with a slow intravenous infusion of dexamethasone, a phosphate ester, to manage wheezing episodes and alleviate respiratory distress [9]. The respiratory symptoms continued to improve on the second day of hospitalization. An otorhinolaryngological evaluation revealed no clinically significant findings.

**Figure 3:**
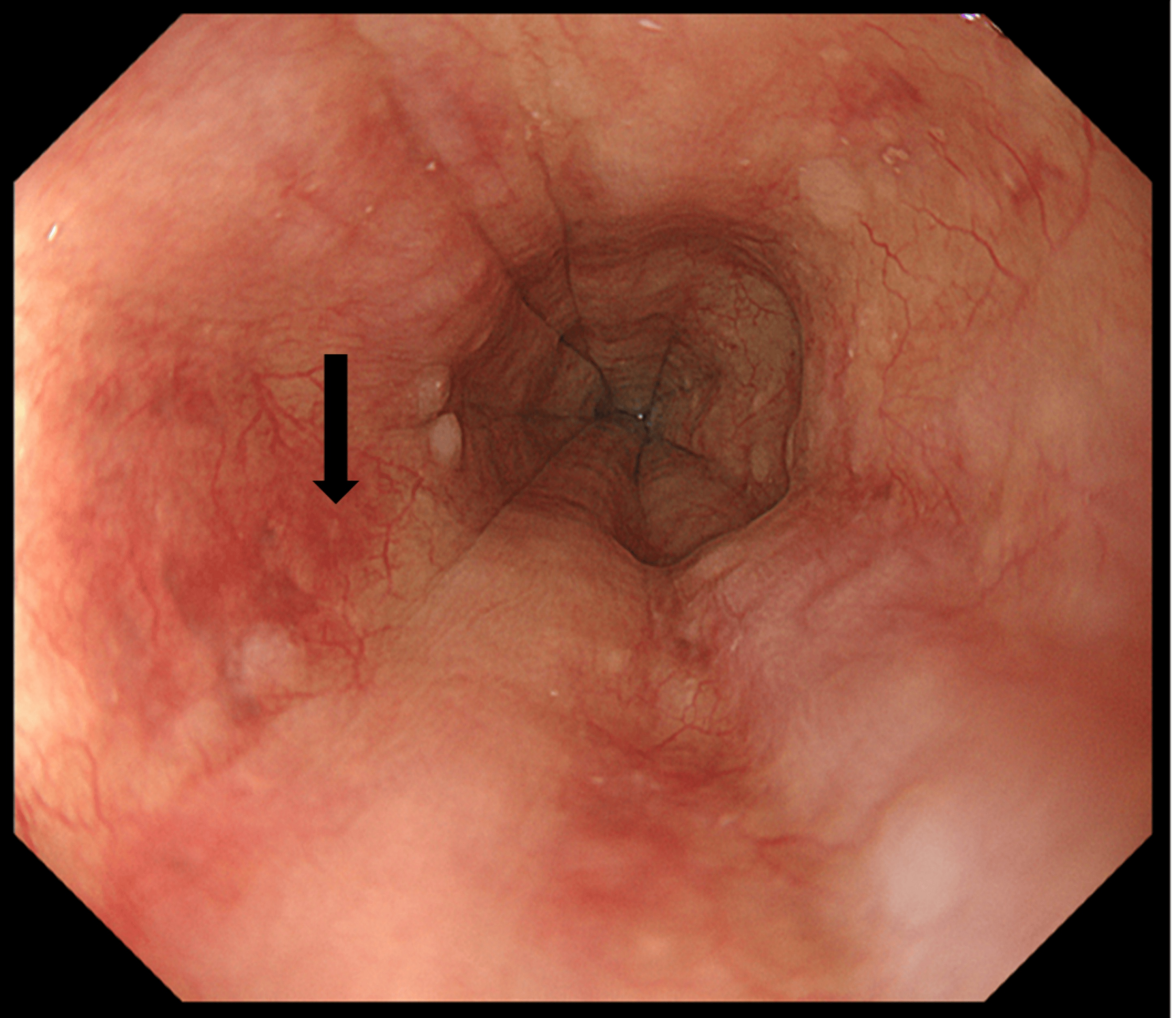
Endoscopic images of the lower esophagus Upper endoscopy showed mucosal edema in the lower esophagus without findings that suggested gastroesophageal reflux disease, longitudinal grooves, or vitiligo. Two mucosal biopsies were taken from areas with severe edema (↓).

**Figure 4:**
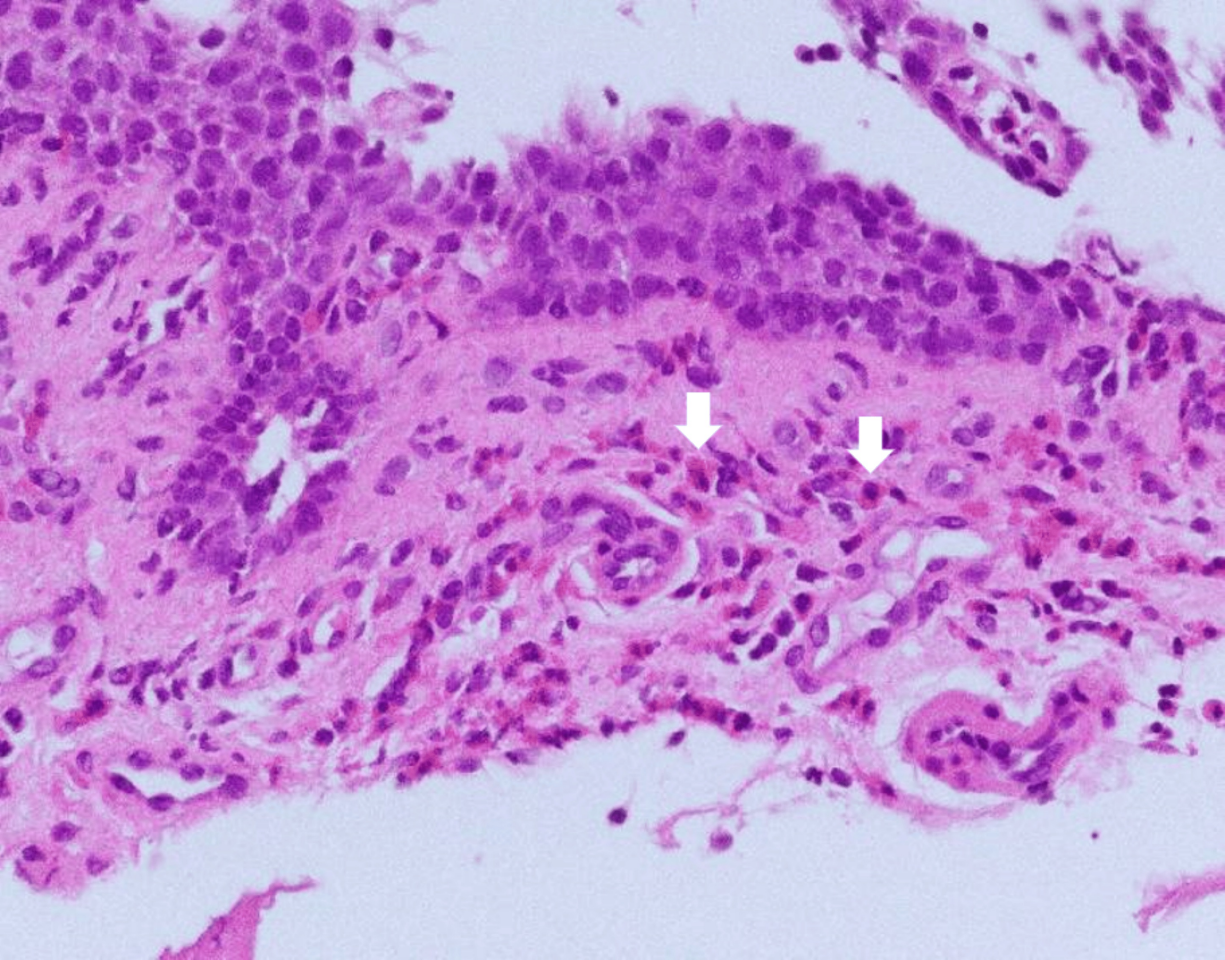
Histopathological image Histopathological examination of the lower esophageal mucosa showed eosinophil infiltration at a rate of 73 per high-power field (reference: <15 per high-power field)(↓).

On the third day of hospitalization, an upper endoscopy revealed mucosal edema in the lower esophagus, but there was no evidence of gastroesophageal reflux disease, longitudinal grooves, or vitiligo (Figure 3). Histological examination of the lower esophageal mucosa showed eosinophil infiltration at a rate of 73 per high-power field (reference: <15 per high-power field) (Figure 4). Accordingly, the patient was diagnosed with EoE [10]. Considering the absence of notable findings in the stomach or duodenum, no biopsies were performed.

The patient began treatment for EoE with swallowed topical ‘inhaled corticosteroids’ [10], and her abdominal symptoms resolved over the course of approximately 1 month. She also continued asthma treatment with fluticasone furoate/vilanterol trifenatate, an inhaled corticosteroid and long-acting beta2-agonist combination. At the 6-month follow-up visit after hospital discharge, she had not experienced any symptom recurrence.

## Discussion

Our patient was simultaneously diagnosed with AERD and EoE after NSAID administration. Although EoE typically develops after AERD onset or during aspirin desensitization therapy [5, 6], our patient was diagnosed with AERD triggered by NSAID administration for epigastralgia in the context of undiagnosed EoE. To our knowledge, this is the first case in which AERD and EoE were simultaneously diagnosed after NSAID administration to a patient with asthma who lacked a history of esophageal diseases.

Our patient’s diagnosis was supported by reports of respiratory distress in patients with AERD who had received 1000-1500 mg of acetaminophen [11]. The severity of AERD symptoms varies among patients; some experience mild asthma, as in our patient, with relatively well-controlled disease; only 81% of patients exhibiting AERD have been diagnosed with nasal polyps [12]. Both AERD and NSAID-induced anaphylaxis are recognized within the spectrum of aspirin-exacerbated diseases [13]. Based on the clinical picture, we judged the patient to AERD with EoE; it is important to note that although an aspirin challenge test increases the accuracy of AERD diagnosis [2], such a test was not conducted here to protect the patient’s health.

Several bioactive substances involved in eosinophil infiltration of the esophagus are implicated in the pathogenesis of AERD and EoE [14-17]. Although the overproduction of prostaglandin D2 (PGD2) has been reported to contribute to the development of AERD, the underlying mechanism remains unclear [14]. Mast cell hyperplasia and activation due to thymic stromal phosphoprotein dysregulation, accompanied by PGD2 release, are involved in AERD [15]. PGD2 also acts as an esophageal chemoattractant for eosinophils in EoE [16]. Additionally, eotaxin-3 has been observed in the esophageal tissue of an EoE patient with AERD [17]. These shared mechanistic pathways suggest an interplay between AERD and EoE, supporting their simultaneous development in our patient.

Although shared mechanistic pathways are known, no prior literature has described AERD and EoE simultaneous diagnosis. Such cases may be rare or easily overlooked in clinical practice due to several factors. First, diagnosing EoE based on clinical symptoms alone can be challenging. Typical EoE symptoms comprise dysphagia/impaction and heartburn/acid reflux; epigastralgia/abdominal pain is atypical (<5% of total cases), and some patients may experience no symptoms (18.8% of total cases) [4]. Endoscopic findings in EoE typically include edema, rings, exudates, furrows, and strictures, but some patients may exhibit normal or non-specific findings [10]. Therefore, multiple biopsies (≥6 specimens) from 2-3 sites are recommended if the esophageal mucosa appears normal on imaging [3]. CT scans can also aid diagnosis, as in our patient; such scans show esophageal wall thickening in approximately half of cases [18]. These complex signs and symptoms may hinder recognition of simultaneous AERD and EoE. Second, the ages of onset for AERD and EoE typically differ: the 30s for AERD [19] and middle age (i.e., 40-60 years) for EoE [4]. Furthermore, a substantial delay (3 months to 6 years) may occur between initiating desensitization therapy for AERD and the diagnosis of EoE [6, 7]. Because AERD and EoE may develop at different times or be misdiagnosed as other clinical entities, no reports exist regarding a simultaneous diagnosis of AERD and EoE. Despite the rarity of such reports, clinicians should be vigilant about the potential for the simultaneous onset of these two diseases.

Standard treatments for AERD include additional drug therapy targeting the leukotriene pathway, avoidance of all NSAIDs, and management according to guidelines for asthma and sinus diseases [20]. Treatments for EoE include elimination diets, topical corticosteroids, systemic corticosteroids, leukotriene antagonists, and endoscopic dilation [10]. Although daily administration of high-dose aspirin to achieve desensitization is effective in patients with AERD [20], clinicians should be aware of its potential to trigger EoE. Other treatments for AERD include cysteinyl leukotriene receptor 1 antagonist, 5-lipoxygenase inhibitors, anti-IgE therapy, and drugs currently in clinical trials (e.g., P2Y12 receptor antagonists, thromboxane receptor antagonists, chemoattractant receptor-homologous molecule expressed on T helper type 2 antagonists, IL-5 inhibitors, and IL-4Rα inhibitors) [20]. Clinicians should be familiar with the clinical presentation and imaging features of EoE, ensure prompt diagnosis and appropriate treatment, and avoid unnecessary NSAID administration.

## Conclusions

We described a patient with simultaneous AERD and EoE diagnoses after NSAID administration. These findings suggest a close interplay between AERD and EoE. Clinicians should consider the potential for coexisting EoE when a patient with epigastralgia develops AERD after taking NSAIDs. Further studies of similar cases are necessary.
